# Time-resolved functional analysis of acute impairment of *frataxin* expression in an inducible cell model of Friedreich ataxia

**DOI:** 10.1242/bio.017004

**Published:** 2016-04-22

**Authors:** Dörte Poburski, Josefine Barbara Boerner, Michel Koenig, Michael Ristow, René Thierbach

**Affiliations:** 1 Institute of Nutrition, Friedrich Schiller University (FSU) Jena, Dornburgerstraße 24, Jena D-07743, Germany; 2 Laboratoire de Génétique de Maladies Rares EA7402, Institut Universitaire de Recherche Clinique, Université de Montpellier, Montpellier F-34093, France

**Keywords:** Frataxin, Friedreich ataxia, Mammalian cell model, Iron sulfur cluster biosynthesis, ROS, HTS

## Abstract

Friedreich ataxia is a neurodegenerative disease caused by a GAA triplet repeat expansion in the first intron of the *frataxin* gene, which results in reduced expression levels of the corresponding protein. Despite numerous animal and cellular models, therapeutic options that mechanistically address impaired frataxin expression are lacking. Here, we have developed a new mammalian cell model employing the *Cre/loxP* recombination system to induce a homozygous or heterozygous *frataxin* knockout in mouse embryonic fibroblasts. Induction of Cre-mediated disruption by tamoxifen was successfully tested on RNA and protein levels. After loss of frataxin protein, cell division, aconitase activity and oxygen consumption rates were found to be decreased, while ROS production was increased in the homozygous state. By contrast, in the heterozygous state no such changes were observed. A time-resolved analysis revealed the loss of aconitase activity as an initial event after induction of complete frataxin deficiency, followed by secondarily elevated ROS production and a late increase in iron content. Initial impairments of oxygen consumption and ATP production were found to be compensated in the late state and seemed to play a minor role in Friedreich ataxia pathophysiology. In conclusion and as predicted from its proposed role in iron sulfur cluster (ISC) biosynthesis, disruption of frataxin primarily causes impaired function of ISC-containing enzymes, whereas other consequences, including elevated ROS production and iron accumulation, appear secondary. These parameters and the robustness of the newly established system may additionally be used for a time-resolved study of pharmacological candidates in a HTS manner.

## Abstract (precis)

**Summary:** The use of a new mammalian cell model with inducible homozygous and heterozygous *frataxin* knockout allows new insights into the chronology and causes of the disease Friedreich ataxia.

## INTRODUCTION

Friedreich ataxia (FRDA) (OMIM #229300) is the most common autosomal recessive inherited ataxia with a prevalence of 1:30,000 to 1:50,000 in Caucasian population (Embirucu et al., 2009). FRDA is caused by a GAA triplet repeat expansion in the first intron of the *frataxin* gene that results in transcriptional silencing of the mitochondrial frataxin protein and therefore reduced expression level of 5-30% (Campuzano et al., 1997, 1996; Koutnikova et al., 1997; Pianese et al., 2004). The number of the GAA repeats can vary between 120-1700 and is inversely correlated with the age of onset and rate of disease progression (Filla et al., 1996; Santoro et al., 1999; Durr et al., 1996). Most of the FRDA patients are homozygous for the GAA expansion and only 2-6% of the patients are compound heterozygous with a GAA expansion on one and another mutation on the other allele (Campuzano et al., 1996; Monros et al., 1997). Over sixty different point, insertion and/or deletion mutations have been found and can influence either *frataxin* stability or its interaction with other proteins (Galea et al., 2015). *Frataxin* mRNA is mainly expressed in tissues with a high metabolic rate (including heart, liver, kidney and brown fat) (Koutnikova et al., 1997; Jiralerspong et al., 1997), whereas the nervous system and heart seem to be the most severely affected tissues (Pandolfo, 2009). FRDA is characterized by a progressive degeneration of the spinal cord and peripheral nerves, which lead to movement disorders, muscle weakness and dysarthria (Parkinson et al., 2013). Besides these neurological symptoms patients often develop a life span reducing cardiomyopathy (Tsou et al., 2011), up to 30% manifest diabetes mellitus of unknown origin (Ristow, 2004) and even associations with increased tumor formation in mice are described (Thierbach et al., 2005). Today's therapeutic strategies to overcome FRDA symptoms include (i) increasing frataxin level (e.g. HDAC inhibitors, erythropoietin) (Rai et al., 2008; Sturm et al., 2005b), (ii) reducing iron mediated toxicity through iron chelators (e.g. deferiprone) (Boddaert et al., 2007; Kakhlon et al., 2008) or (iii) improving the cellular antioxidant defense (e.g. idebenone, PPARγ agonists, Nrf2 inducers) (Marmolino et al., 2010; Hausse et al., 2002; Mariotti et al., 2003; Shan et al., 2013). Until now, no generally approved therapy for FRDA exists that cures or even slows the disease (Wilson, 2012; Santos et al., 2010; Mancuso et al., 2010), and we still do not fully understand the underlying disease mechanisms. Furthermore, the precise function of the protein frataxin remains unclear, but the involvement of frataxin in the synthesis of iron-sulfur clusters (ISCs) and ISC-containing proteins is generally accepted (Gerber et al., 2003; Muhlenhoff et al., 2002; Schmucker et al., 2011; Stehling et al., 2004; Rouault, 2012). Several studies in yeast, mice or FRDA patients support the role of frataxin in ISC-synthesis and showed that frataxin deficiency leads to a reduced aconitase activity (Al-Mahdawi et al., 2006; Rotig et al., 1997), respiration (Wilson and Roof, 1997; Zarse et al., 2007) and generation of mitochondrial ATP (Lodi et al., 1999; Thierbach et al., 2005) as well as an increase of mitochondrial iron (Babcock et al., 1997; Puccio et al., 2001) and oxidative stress (Ristow et al., 2003; Vazquez-Manrique et al., 2006). Conversely, an overexpression of frataxin in mammalian cells revealed an increase in respiration and ATP content (Ristow et al., 2000; Schulz et al., 2006). However, we still do not know exactly which metabolic consequences primarily occur after frataxin depletion and will be most relevant for further disease therapy strategies.

Frataxin is evolutionary highly conserved from the prokaryote *Escherichia* *coli*, the unicellular eukaryote *Saccharomyces* *cerevisiae* to multicellular non-mammalian (*Caenorhabditis* *elegans*, *Drosophila melanogaster*, *Arabidopsis thaliana*) and mammalian (*Mus* *musculus*) organisms (Koutnikova et al., 1997; Ventura et al., 2006; Busi et al., 2004; Canizares et al., 2000; Gibson et al., 1996). Generating suitable models to understand the underlying disease mechanisms are challenging and not all models show the specific symptoms or biochemical features associated with FRDA (Martelli et al., 2012). Since cells from FRDA patients do not spontaneously exhibit the ISC enzyme deficiency (Calmels et al., 2009b), the development of mammalian cellular models is especially required to understand cellular consequences after a frataxin deficit. This necessity is emphasized by the recent publication of a new cellular model of Vannocci et al. (2015). Although they used the already transformed HEK-293 cells, they created a model with an inducible exogenous *frataxin* gene which rescues the cells from the homozygous knockout of the endogenous *frataxin* gene. Nevertheless, to discover new therapeutic approaches we still require stable FRDA models that reproduce the primary events after frataxin depletion and enable us to screen potential pharmacological substances.

Here, we present a new inducible mammalian cell model for FRDA that shows typical features of the disease and gives us the opportunity to monitor their alterations over time. By using the *Cre/loxP* recombination system (Ristow et al., 2003; Brightbill et al., 2015) in murine embryonic fibroblasts, we are able to create a homozygous or heterozygous knockout of the *frataxin* gene at a specific time and therefore reduce mitochondrial frataxin protein, respectively. By characterizing the consequences after a frataxin deficit in our cell model, we found alterations in cell division, aconitase activity, ATP and iron content, ROS production and oxygen consumption. Additionally we can show how these known metabolic parameters vary in time and therefore identify early and late events during the frataxin disruption process. This mammalian FRDA model can be used for time-resolved analysis of pharmacological drugs and their effect on metabolic parameters in a HTS manner. These findings will help us to better understand the disease mechanisms and opens up new points of action in FRDA treatment.

## RESULTS

### Establishing the *frataxin* knockout system

**Fig. 1.**
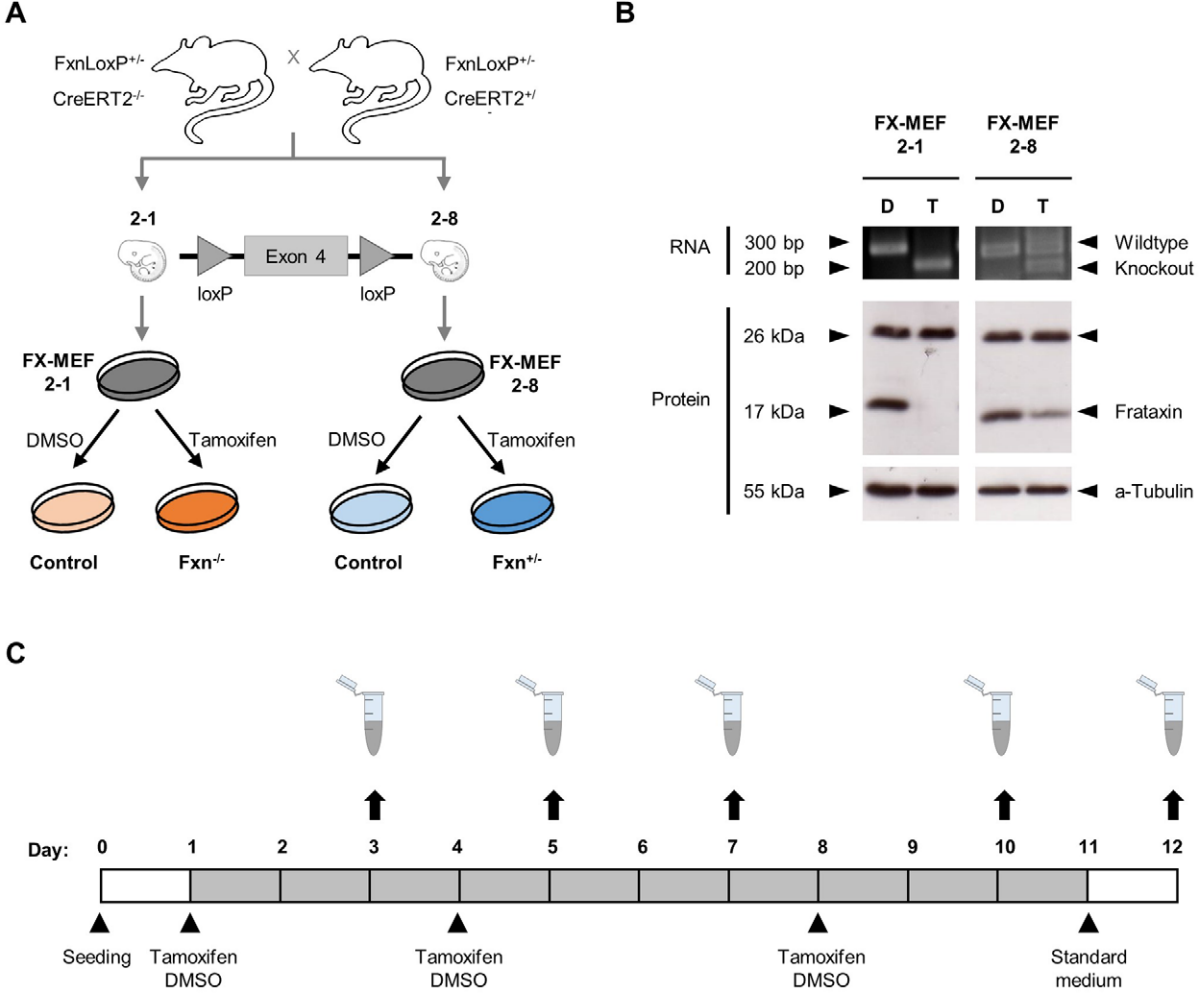
**Establishment of the homozygous and heterozygous *frataxin* knockout system.** (A) C57BL/6J mouse strains with a loxP-flanked exon 4 of the *frataxin* gene and a tamoxifen-inducible Cre-recombinase (CreER^T2^) were crossed and several MEF cell lines isolated. After selection by genotype and growth manner the FX-MEF 2-1 (Fxn^−/−^) and FX-MEF 2-8 (Fxn^+/−^) cell line was finally chosen. By adding 1 µM tamoxifen into the culture medium, a stable homozygous or heterozygous knockout is achieved. (B) Successful *frataxin* knockout at RNA and protein level after treatment with 1 µM tamoxifen (T) or DMSO (D) for 48 h was verified with reversed transcription PCR (primers located in exon 3 and 5 of the *frataxin* gene) and immunoblot. α-Tubulin served as loading control. (C) Treatment plan, with changes of medium supplemented with 1 µM tamoxifen or DMSO on day 1, 4 and 8 after seeding (split when needed). For all endpoint experiments on day 12 a change to standard medium was performed on day 11. Time response monitoring of aconitase activity, ROS and ATP formation, iron content and oxygen consumption took place on day 3, 5, 7 and 10, while growth manner was obtained by daily fixation.

To generate our new *frataxin* knockout model we crossed C57BL/6J mouse strains heterozygous for a loxP-flanked exon 4 of the *frataxin* gene and either heterozygous or without a tamoxifen-inducible Cre recombinase (CreER^T2^). Afterwards we isolated several strains of murine embryonic fibroblasts (MEF) and performed a selection by genotyping and growth manner to finally pick two cell lines with an inducible homozygous (FX-MEF 2-1) and heterozygous (FX-MEF 2-8) knockout. To establish a complete knockout eventually, 1 µM tamoxifen was admitted to the culture medium and DMSO as control (Fig. 1A).

Efficiency of the knockout at transcriptional and translational levels were proved by using reverse transcribed PCR, with primers located in exons 3 and 5 of the *frataxin* gene and immunoblotting against murine frataxin protein (Fig. 1B). *Frataxin* knockouts were efficient on RNA level, shown by a smaller transcript in the tamoxifen treated homozygous FX-MEF 2-1 cells and a wildtype as well as knockout band for the heterozygous FX-MEF 2-8. We also detected a total disruption of the frataxin protein in the tamoxifen treated FX-MEF 2-1 cells and an only slightly reduced expression in the FX-MEF 2-8.

We established a defined pattern of treatment with three doses of tamoxifen or DMSO on day 1, 4 and 8 after seeding (Fig. 1C), as cultivation of the tamoxifen-treated FX-MEF 2-1 cells lead to a strong growth inhibition and further death of the cells after 14 days.

### Disruption of *frataxin* leads to fundamental metabolic changes

**Fig. 2.**
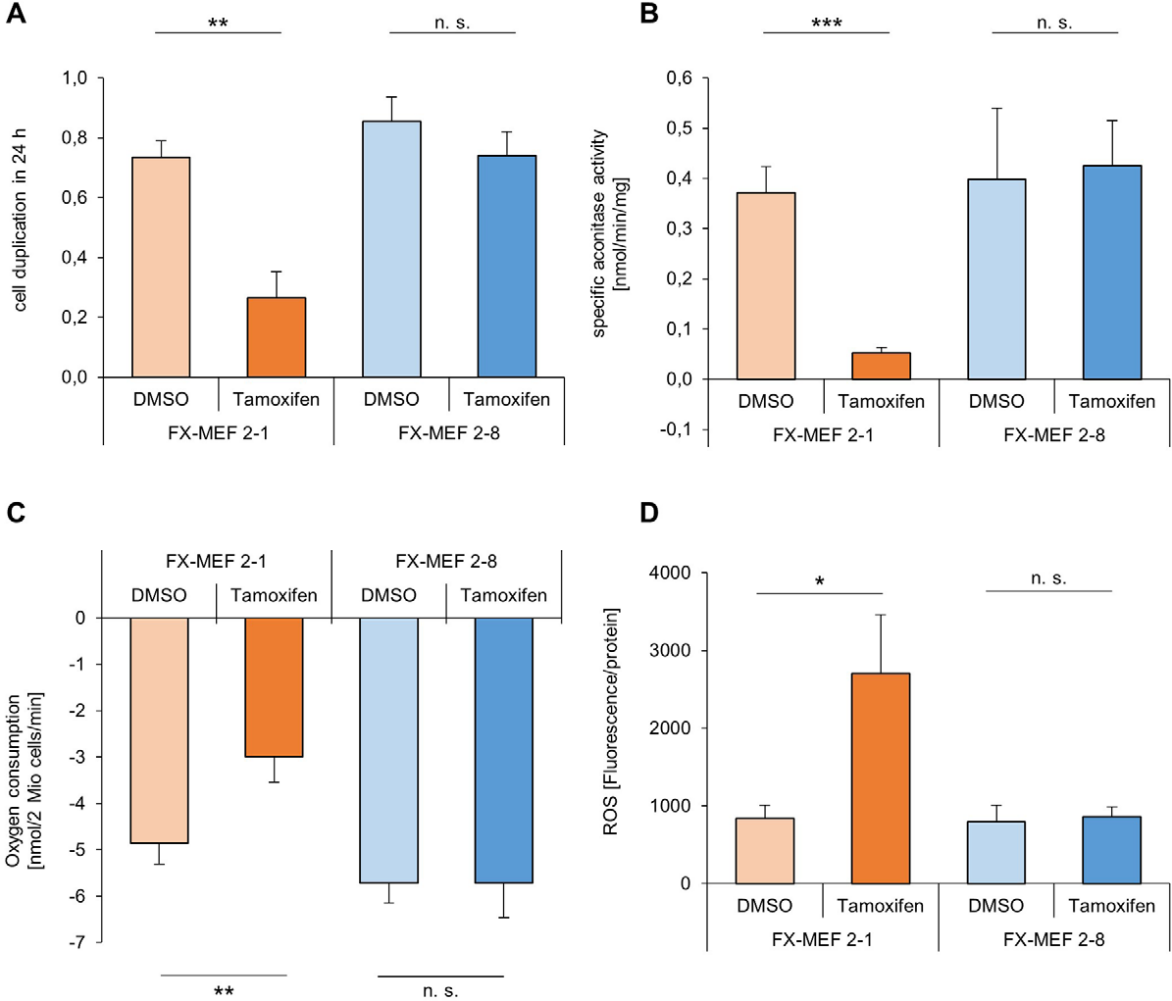
***Frataxin* knockout initiates several metabolic alterations after 12 days of treatment.** After incubation of the FX-MEF 2-1/2-8 cells with 1 µM tamoxifen or DMSO, endpoint measurements were accomplished on day 12. Results indicated are mean±s.d. for three independent experiments. Statistical differences are displayed as *P<0.05; **P<0.01; ***P<0.001 and not significant (n.s.) according to a two-sample Student's *t*-test (unequal variances). (A) Cells were fixed with 10% trichloroacetic acid and stained every day using the sulforhodamin B protocol. The linear slope was used to calculate the number of duplications per 24 h. (B) Aconitase activity was detected spectrometrically by monitoring the formation of NADPH over 60 min. The linear slope was calculated as specific enzyme activity (nmol/min/mg). (C) Oxygen consumption was measured using a Clark-type electrode. 2 Mio cells/ml were applied and the change of oxygen consumption monitored over 5 min. (D) ROS was measured as fluorescence intensity of the MitoTracker Red CM-H_2_X dye after 30 min of incubation.

We initially performed endpoint measurements of growth manner, aconitase activity, ROS formation and oxygen consumption on day 12 to characterize our cell system, as alterations of several metabolic parameters after *frataxin* disruption are described in many different model organisms as well as FRDA patients. We observed that tamoxifen treatment and total disruption of the frataxin protein leads to a significant growth inhibition of 64% in the homozygous FX-MEF 2-1 cells on day 12 (Fig. 2A). To address metabolic consequences of a reduced expression of *frataxin* we first determined the aconitase activity as an indicator of ISC-dependent proteins. The enzyme aconitase catalyzes the conversion of citrate to isocitrate in the citric acid cycle and needs for its activity an intact [4Fe-4S]^2+^ cluster. A homozygous knockout of *frataxin* leads to a significant reduction of aconitase activity, remaining by 14% compared to control (Fig. 2B). We further investigated potential effects on the respiratory chain (also depending on ISCs), because it is important for cellular energy conversion and it is also a possible site for electron leakage and increased production of reactive oxygen species (ROS). An induced total *frataxin* knockout by tamoxifen leads to a clearly diminished oxygen consumption (Fig. 2C) and an additional large increase of ROS (Fig. 2D). Cells with a heterozygous knockout (FX-MEF 2-8) exhibit a 13% decrease in growth manner compared to control and show no significant change in aconitase activity as well as ROS production or oxygen consumption (Fig. 2A-D).

### Dissecting of early versus late events after loss of frataxin function

**Fig. 3.**
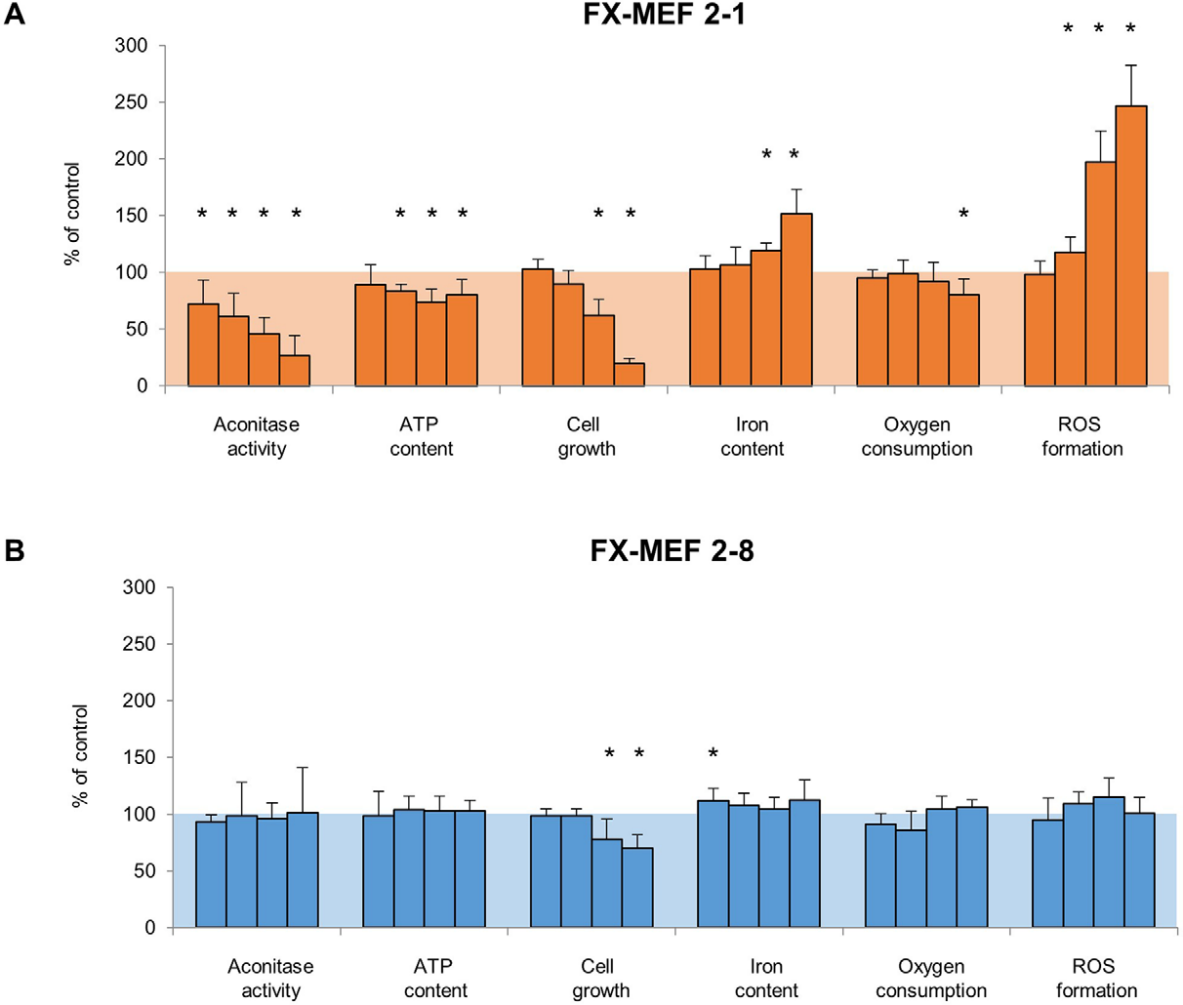
**Early and late consequences after *frataxin* disruption.** FX-MEF 2-1 and FX-MEF 2-8 cells were treated according to the standard treatment pattern with 1 µM tamoxifen or DMSO. Parameter measurements were performed on days 3, 5, 7 and 10 (illustrated as bars) to monitor the impact of a homozygous (orange) or heterozygous (blue) knockout over time. Displayed are the relative differences in percent of the measured parameter compared to the DMSO control (100%) and indicated as mean±s.d. for six independent experiments. Statistical analyses were accomplished with a Wilcoxon signed-rank test on the basis of the absolute values. Significant differences between knockout and control are displayed as *P<0.05.

Because frataxin function and the following metabolic consequences after total disruption are still not fully understood, we tried to monitor these parameters (with additional cellular ATP level and iron content) and their variations in time up to day 10 in our cell model. Measurements for oxygen consumption in the homozygous state showed a late and weak reduction, significant only at day 10, and seemed to be regulated very well (Fig. 3A). In addition we found a weak decrease in ATP production starting at day 5, but this effect did not strengthen over time. Cell duplication of the *frataxin*-disrupted cells diminished constantly during the experimental setup, being significant on day 7 and 10. More substantial alterations were observed as a significant increase in iron content on day 7 and 10 as well as a steady enhancement in ROS production beginning at day 5. The first recognizable event after loss of frataxin function seemed to be the significant decline of aconitase activity, which starts already at day 3 and further continues until day 10. A look at the heterozygous state reveals a totally different picture (Fig. 3B). Aconitase activity, ATP level, oxygen consumption as well as ROS production were not significantly altered after tamoxifen treatment and partial loss of frataxin function. Unexpectedly, we observed a slight increase in iron content of the cells at day 3 which normalized for all other time point measurements and seemed to be of no great relevance. Although the cellular consequences of a heterozygous knockout could be compensated for most of the measured parameters, we also recorded the aforesaid growth inhibition (starting on day 7), but in a clearly lower degree in comparison to the homozygous FX-MEF 2-1 knockout cells.

## DISCUSSION

The molecular cause of the neurodegenerative disease Friedreich ataxia is the reduction of the mitochondrial protein frataxin below a critical level. The essential function of frataxin beyond the involvement in the ISC assembly machinery is still not fully understood. Although only humans or primates contain a GAA repeat in the *frataxin* gene intron 1 sequence (Montermini et al., 1997) and frataxin mouse models are more appropriate to evaluate tissue-specific disease features, we still require suitable cellular models for FRDA research which imitate biochemical features in a reduced complexity. Especially mammalian cellular *frataxin* knockout models are of great value for investigating the underlying molecular disease mechanisms or screening potential drug candidates (Perdomini et al., 2013). Several approaches like a ribozyme antisense strategy or RNAi have been carried out over the past years to reduce levels of *frataxin* in a wide range of cell systems like murine fibroblasts, T-Rex-293, HEK-293 and HeLa cells (Stehling et al., 2004; Lu and Cortopassi, 2007; Calmels et al., 2009a,b; Vannocci et al., 2015). In order to overcome the lethal phenotype of a homozygous disruption, a transfection with an exogenous murine or human *frataxin* gene was established successfully. However, not all cell models resembling the biochemical consequences of the human disease have been studied in a time-course manner or are stable over a long time.

In this study we established a novel cellular model of murine fibroblast with the ability to switch off *frataxin* transcription by using the *Cre/loxP* recombination system; this way we generated a homozygous cell line (FX-MEF 2-1) with a complete *frataxin* deficit and a heterozygous control line (FX-MEF 2-8) mimicking only a partial loss of frataxin protein. Long maintenance of the frataxin-depleted fibroblasts revealed a strong growth inhibition consistent to earlier observations of embryonic lethality in *frataxin* knockout mice (Cossee et al., 2000) or RNAi-based human and murine cell models (Calmels et al., 2009b; Stehling et al., 2004). Therefore we developed a specific pattern of treatment and did not extend our experiments over a critical point of 12 days.

The generally accepted function of frataxin is the participation in ISC assembly confirmed by reduced activities of ISC-containing proteins (Muhlenhoff et al., 2002; Lill, 2009; Ye and Rouault, 2010). The citric acid cycle enzyme aconitase with an [4Fe-4S]^2+^ cluster as well as the respiration complexes I-III were found to be reduced in FRDA lymphoblasts (Heidari et al., 2009), mouse models (Puccio et al., 2001; Thierbach et al., 2005) and yeast knockout strains (Rotig et al., 1997; Bulteau et al., 2004). Lu and Cortopassi further observed in a human *frataxin* RNAi model that the first consequence after *frataxin* deficiency seems to be a defect in cytosolic aconitase (Lu and Cortopassi, 2007). In our mammalian cell system we could detect that after full *frataxin* knockout the aconitase activity reduces constantly over time to a minimum level of 14%. Further disturbances in energy metabolism were determined as decreased ATP production and oxygen consumption due to putative respiratory chain impairment. In addition to the loss of ISC protein activity we were able to identify the aconitase reduction as an initial event after *frataxin* disruption, whereas respiratory chain events seem to be a secondary outcome. The primary effect of the decreased ISC protein activity in *frataxin* deficient cells supports the role of frataxin in ISC synthesis (Muhlenhoff et al., 2002; Gerber et al., 2003) and indicates the relevance for the human disease. On the contrary, a partial deficit of *frataxin* did not result in any differences in aconitase activity or respiration complexes over time and suggests that there are only cellular alterations below a critical threshold.

The role of iron in the pathophysiological process of FRDA is still a controversial and much-discussed aspect. For quite a long time mitochondrial iron accumulation was stated as a hallmark of *frataxin* deficiency, supported by iron deposits in patient tissues (Bradley et al., 2000) or yeast lacking YFH1 (Babcock et al., 1997; Muhlenhoff et al., 2002). In contrast, no alterations in cellular iron were observed in human FRDA lymphoblasts and fibroblasts (Sturm et al., 2005a) as well as several cellular *frataxin* knockdown models (Calmels et al., 2009b; Lu and Cortopassi, 2007; Stehling et al., 2004). Another interesting aspect found by the group of T.A. Rouault was the observation of an additional cytosolic iron depletion in cells from FRDA patients, which may further contribute to a reduced *frataxin* transcription and worsening of the disease (Li et al., 2008). However, we were able to detect an increase in cellular iron content after *frataxin* depletion. In accordance to results from a FRDA mouse model published by Puccio et al. (2001) we identified the iron accumulation as a late event and not as an initial factor in FRDA pathogenesis.

The involvement of frataxin in iron metabolism lead to the hypothesis of a vicious cycle in which elevated levels of iron induce the formation of ROS via Fenton reaction and thereby are responsible for the loss of ISC. Observations in a yeast *frataxin* knockout model in R. Lill's group showed that these cells accumulate mitochondrial iron, are less sensitive to oxidative stress and have a decrease in ISC proteins (Muhlenhoff et al., 2002). Occurrences of oxidative insults in DNA of FRDA patients (Schulz et al., 2000) and oxidative sensitivity in FRDA fibroblasts (Sturm et al., 2005a; Wong et al., 1999) as well as organisms like *Caenorhabditis elegans* and *Drosophila melanogaster* (Vazquez-Manrique et al., 2006; Llorens et al., 2007) strengthened the idea of a vicious cycle. Further evidence for the role of ROS in FRDA pathogenesis provided beneficial effects of antioxidants like idebenone on the cardiac symptoms of FRDA patients and in a murine FRDA cardiomyopathy (Frda/MCK) model (Hausse et al., 2002; Mariotti et al., 2003; Seznec et al., 2004). Otherwise, Seznec and colleagues could show in the Frda/MCK mice that oxidative stress seems to play a minor role in the course of the disease (Seznec et al., 2005). Based on these different observations we wanted to further question the vicious cycle hypothesis and investigated all three events in our newly established *frataxin* knockout model in parallel. A full depletion of *frataxin* shows a massive increase in ROS production, but this event takes place after ISC interference (shown as aconitase activity) and even before iron accumulation. Hence, iron accumulation seems not to be the cause of oxygen species in our cell system and rather the impaired respiration chain (measured as simultaneously diminished ATP level and late reduced oxygen consumption) is responsible for the ROS appearance. Furthermore, the loss of aconitase activity is most likely caused by decreased ISC synthesis due to *frataxin* knockout, than by elevated ROS level. With our investigations we agree with the revisited vicious circle hypothesis by A. Bayout et al., that iron accumulation is a late event and an abnormal oxidative status is one of the first consequences after *frataxin* deficiency (Bayot et al., 2011).

The newly presented inducible *frataxin* knockout model reproduces biochemical consequences of the human FRDA disease und gives us the opportunity to have a look on time-dependent effects after *frataxin* impairment in a stable mammalian cell system. As a result of the defined treatment pattern we are able to measure six different parameters of interest in parallel. The model indicates that after *frataxin* depletion the first cellular effect is on ISC-containing proteins, followed by secondary events like ROS and ATP production and an even later iron accumulation. These observations need to be taken into account for developing new therapeutic strategies for this still untreatable disease. Furthermore, this murine FRDA model might be useful for testing new pharmacological candidates to delay or cure the metabolic features following loss of frataxin function and helps us to get a first impression, on how they might influence FRDA pathogenesis.

## MATERIALS AND METHODS

### Generation of the *frataxin* knockout cells

The CreERT2 mice (Seibler et al., 2003) were generated as described by Artemis Pharmaceuticals and the frataxin loxP mice (Puccio et al., 2001) were generated and maintained as described before, both on a C57BL/6 background for several generations. Care of the mice and experimental proceduers were performed in accordance to the relevant laboratory animal regulations. MEF cells were isolated from mouse embryos according to a protocol by Xu (2005). Briefly, mice were euthanized and the uterus was removed. After washing the uterus with PBS all embryos were dissected out intact and liver, heart, brain and eyes were removed and used for genotyping. Embryos were cut into small pieces, transferred to a 50 ml tube and 2 ml of ice-cold 0.25% trypsin-EDTA was added for 5 min on ice. Afterwards tubes were incubated for 15 min in a 37°C water bath and 5 ml of MEF culture medium was added. To break up the digested tissues into a cell suspension repeatedly pipetting up and down was necessary. Cell suspension was finally plated in a 10 cm culture dish in MEF culture medium. Primary MEF cells underwent serial passaging and became immortalized by passing their growth-crisis stage.

### Culture conditions of the FX-MEF cells

The immortalized MEF cells (FX-MEF) were cultured in DMEM (Dulbecco's modified Eagle's medium) containing 4.5 g/l D-glucose and 10% fetal bovine serum and tested routinely for contamination. Cells were treated with 1 µM 4-hydroxytamoxifen (Cat.: H7904, Sigma-Aldrich) to generate the homozygous or heterozygous knockout and the same amount DMSO as control. All experiments were accomplished with a defined pattern of handling with a duration of 12 days and medium changes on day 1, 4, 8 (with tamoxifen or DMSO) and day 11 (standard medium). Measurements for the time response monitoring took place on day 3, 5, 7 and 10.

### RNA isolation and amplification

Total RNA was isolated following the TRIzol instruction manual (Cat.: 15596-026, Life Technologies). Measuring the absorbance at 260 nm and 280 nm was used to determine the RNA concentration and quality (A_260/280_>1.8). Reverse transcription PCR was performed as previously described (Puccio et al., 2001) using primers 5′-CACTTGGATCCTCTAGACGAGACAGCG and 5′-TTTAGTCAGCTCCCTGGCC, located in exon 3 and exon 5 of the *frataxin* cDNA.

### Protein extraction and immunodetection

Protein samples were prepared by lysing (Cell Signaling Technologies lysis buffer) and sonicating (Bandelin Sonopuls, Berlin, Germany) of the cells and quantified according to Bradford's method (Bradford, 1976). SDS-PAGE was performed with a 16% gel and 30 µg protein extract per lane. The separated proteins were transferred to a PVDF membrane by semi-dry western blotting, followed by incubation with different antibodies. Detection of the knockout at translational level was achieved with a polyclonal antibody against mouse frataxin (Puccio et al., 2001) and an additional monoclonal antibody against α-Tubulin (1:3000, Cat.: T9026, Sigma-Aldrich).

### Growth curve

The sulforhodamin B assay (Skehan et al., 1990) was used to evaluate the cell growth and density during the experiments. Therefore cells were fixed for 45 min at 4°C with 10% trichloroacetic acid, stained with sulforhodamin B for 15 min at room temperature and washed repeatedly with 1% acetic acid. By adding alkaline 10 mM Tris buffer (pH 10.3) the protein-bound dye was extracted and optical density was measured at 560 nm.

### Aconitase activity assay

Aconitase activity was determined spectrometrically by monitoring the formation of NADPH at 340 nm. The assay mixture contained 50 mM Tris-HCl (pH 7.4), 60 mM sodium citrate, 1 mM MnCl_2_, 20 mM NADP^+^, and 4 units/ml of isocitrate dehydrogenase. 130 µg of the protein extract was filled up to 150 μl with 50 mM Tris-buffer (pH 7.4) and loaded into a 96-well plate. By adding 150 μl of the assay mixture the enzyme reaction was started and the change of absorbance at 340 nm was measured for 60 min at 37°C. The aconitase activity was calculated from the slope of the linear portion.

### Determination of ROS and ATP

To quantify the ROS and ATP content, cells were seeded in a 96-well plate and handled following the standard pattern of treatment. The fluorescence intensity (Ex 579 nm/Em 599 nm) of mitochondrial derived ROS were assessed with the fluorescent dye MitoTracker Red CM-H_2_XRos (Cat.: M-7513, Life Technologies) after incubation of the cells with 1 µM staining solution for 30 min. ATP content of the cells was quantified with the CellTiter-Glo Luminescent Cell Viability Kit (Cat.: G7571, Promega). Protein content was determined by the bicinchoninic acid assay (ROS) (Smith et al., 1985) or sulforhodamin B (ATP) as reference value.

### Oxygen consumption

Oxygen consumption was measured by using a Clark-type electrode (Hansatech Instruments; Norfolk, UK). Therefore cells were washed, trypsinised and counted with a Neubauer chamber. A solution of 2 Mio cells/ml was filled into the air-tight and 37°C tempered Clark electrode chamber to monitor the respiration rate for 5 min. Available oxygen in the chamber passes through the Teflon membrane to reduce the platinum cathode, meanwhile the silver anode is oxidized. The produced current by the electron shifting was used to calculate the respiration rate.

### Cellular iron content

Cells were washed, trypsinized and adjusted to the same protein amount using the BCA assay (Smith et al., 1985). Quantification of total cellular iron content was performed according to a colorimetric method of Fish (1988). Protein bound iron was released by incubating the samples for 2 h at 60°C with 0.285 M potassium permanganate/1.2 M hydrochloric acid. Afterwards assay solution (6.5 mM ferrozine, 13.1 mM neocuproine, 2 M ascorbic acid, 5 M ammonium acetate) was admitted for 15 min and samples were centrifuged (5 min, 6600×***g***) to remove precipitates. Formation of the magenta-colored Fe(II)-ferrozine complex was measured at 562 nm.

### Statistical analysis

Calculations of statistical differences of the endpoint experiments on day 12 were assessed according to a two sample Student's *t*-test (unequal variances). The Wilcoxon signed-rank test was used for statistical analyses of the time response monitoring and a probability value of *P*<0.05 was considered to be statistically signiﬁcant. Analyses were performed using Microsoft Excel™ and IBM SPSS Statistics 22.
